# VEGF and SEMA4D have synergistic effects on the promotion of angiogenesis in epithelial ovarian cancer

**DOI:** 10.1186/s11658-017-0058-9

**Published:** 2018-01-03

**Authors:** Ying Chen, Lei Zhang, Wen-xin Liu, Ke Wang

**Affiliations:** 1 Department of Gynecologic Oncology, Tianjin Medical University Cancer Institute and Hospital, Huanhuxi Road, Hexi District, Tianjin, 300060 China; 2 Key Laboratory of Cancer Prevention and Therapy, Tianjin, 300060 China; 3 National Clinical Research Centre of Cancer, Tianjin, 300060 China

**Keywords:** SEMA4D, Vegf, Angiogenesis, Tumor growth, Epithelial ovarian cancer

## Abstract

**Background:**

Anti-angiogenesis therapy that targets VEGF is one of the important treatment strategies in advanced ovarian cancer. However, depending on the pharmaceutical agent, treatment can have undesirable side effects. SEMA4D has recently gained interest for its role in promoting angiogenesis. Here, we try to further understand the mechanism by which SEMA4D promotes angiogenesis in ovarian cancer.

**Methods:**

Correlation and western blot assaya were used to detect the relationship between VEGF and SEMA4D in clinical tissues and cells. Vasculogenic mimicry and transwell migration analyses were used to detect the roles of VEGF, SEMA4D and plexin-B1 on vasculogenic mimicry and migration. Vascular density and SEMA4D expression was determined using immunofluorescence staining in clinical tissues of EOC. Western blot was used to detect the expressions of CD31, MMP2 and VE-cadherin. We also analyzed the relationship between VEGF-SEMA4D and malignant tumor prognosis.

**Results:**

We found that knockdown of VEGF could suppress SEMA4D expression and that the expressions of VEGF and SEMA4D have a positive correlation in EOC cancer tissues. Vasculogenic mimicry and transwell migration analyses showed that SEMA4D and VEGF have a synergistic effect on the promotion of angiogenesis in A2780 and HUVEC cells. Soluble SEMA4D (sSEMA4D) could promote VM and migration in A2780 and HUVEC cells via the SEMA4D/plexin-B1 pathway, but the effect was not noted in stably transfected shR-plexin-B1 cells. In clinical tissues of EOC, the vascular density and SEMA4D/plexin-B1 expression were higher. When VEGF, SEMA4D and plexin-B1 was knocked down, the expression of CD31, MMP2 and VE-cadherin, which are the markers and initiators of angiogenesis and the epithelial–mesenchymal transition (EMT) process were reduced. VEGF and SEMA4D had a positive correlation with the malignant degree of ovarian cancer, and SEMA4D can serve as an independent prognostic factor.

**Conclusions:**

VEGF and SEMA4D have synergistic effects on the promotion of angiogenesis in epithelial ovarian cancer. Targeting VEGF and the SEMA4D signaling pathway could be important for the therapy for EOC.

## Background

Epithelial ovarian cancer (EOC) is the most common malignant gynecological tumor. The EOC mortality rate ranks first among the gynecological malignancies and the overall 5-year survival rate is only ~30% [1]. As with most malignant solid tumors, the rich microvascular environment encourages the growth of EOC and the prognosis is generally poor. The formation of large numbers of microvessels is the basis for the growth and metastasis of ovarian cancer in general and EOC in particular [2].

Vascular endothelial growth factor (VEGF) plays a critical role in the formation of vessels under both physiological and pathological conditions. VEGF increases the formation of nascent blood vessels by promoting the proliferation and migration of endothelial cells, increasing vascular permeability, and inhibiting endothelial cell apoptosis [3, 4].

Angiogenesis is the process of generating new blood vessels from existing blood vessels. In 1971, Folkman proposed that the growth and metastasis of tumors are dependent on tumor angiogenesis [5]. Vascular growth has two different phases: slow and rapid. Without angiogenesis, the primary tumor does not grow. Tumor blood vessels provide the necessary oxygen and nutrients to the tumor tissue, allowing it to grow rapidly, and also facilitate distant metastases [6–8]. Inhibiting angiogenesis is a relatively new anti-tumor strategy [9]. The role of tumor angiogenesis in the development of cancer has also attracted the attention of scholars both at home and abroad [10].

Anti-angiogenesis therapy, mainly targeting anti-VEGF, is an important treatment strategy in numerous malignancies [11]. The targeted therapy bevacizumab (Avastin) inhibits tumor progression by binding to VEGF and blocking its biological activity. However, its clinical effect is not satisfactory. For example, Phase III clinical trials GOG218 [12] and ICON7 [13] showed that chemotherapy plus bevacizumab did not significantly improve overall survival for patients with ovarian cancer after first-line treatment despite the slight increase in progression-free survival. Fischer et al. [14] suggest that once patients with advanced cancer are administered anti-angiogenic therapy, the expression of other pro-angiogenic factors (e.g., SDF-1, FGF-1, FGF-2, etc.) is significantly increased, eventually leading to the failure of anti-VEGF treatment. Therefore, it is important to explore the molecular mechanism of anti-VEGF treatment failure to improve the efficacy of treatments for epithelial ovarian cancer.

The semaphorin superfamily is a class of proteins sharing a common Sema region. One semaphorin, axon guidance protein, which is part of the SEMA family, participates in axon guidance, spindle formation and nerve signal transduction pathways. It was initially discovered in the embryonic nervous system. Recent studies indicate that members of the SEMA family are also involved in the process of tumor angiogenesis [15–17].

The SEMA IV subfamily, a subcategory of the SEMA family, promotes angiogenesis. One important member is semaphorin4D (SEMA4D), which mediates endothelial cell chemotaxis and promotes the process of microvascular formation and distant metastasis in head and neck squamous cell carcinoma [18]. Basile et al. [19] reported that membrane-type matrix metalloproteinase 1 (MT1-MMP) is secreted into the tumor microenvironment and acts in concert with SEMA4D to promote tumor angiogenesis, thus also promoting distant metastasis. Plexins are the only membrane-bound receptors of SEMA4D. Plexin-B1 combines with SEMA4D secreted into the microenvironment from tumor cells to promote tumor angiogenesis and endothelial cell migration and vessel formation [18–20].

Here, we mainly explore the role of SEMA4D in the process of angiogenesis in epithelial ovarian cancer. VEGF and SEMA4D expression exhibit a positive correlation in clinical tissues. Vasculogenic mimicry (VM) and transwell migration analyses showed the synergistic promotional effect of SEMA4D and VEGF on angiogenesis in A2780 and HUVEC cells. Soluble SEMA4D (sSEMA4D) promotes VM and migration in A2780 and HUVEC cells via the SEMA4D/plexin-B1 pathway. This effect was not noted in stably transfected shR-plexin-B1 cells. In EOC tissues, where the vascular network is denser, increased SEMA4D/plexin-B1 expression can be observed. When VEGF, SEMA4D and plexin-B1 were knocked down, the expressions of CD31, MMP2 and VE-Cadherin, which are the markers and initiators of angiogenesis and epithelial–mesenchymal transition (EMT), were reduced. VEGF and SEMA4D were positively correlated with the malignant degree of ovarian cancer. SEMA4D can serve as an independent prognostic factor.

In conclusion, SEMA4D may be an important molecule for anti-angiogenesis therapy, and further development of anti-SEMA4D therapy may give rise to a significant breakthrough in the field of EOC therapy.

## Methods

### Cell lines and cell culture

Human umbilical vein endothelial cells (HUVECs, ScienCell, Cat. #8000) were cultured in endothelial cell medium (ECM, Cat. #1001). The human ovarian cancer cell line A2780 was cultured in DMEM as the basal medium with 10% fetal bovine serum (FBS) and double antibiotics added for cell culture.

### Transwell migration analysis

The transwell migration assay was performed in a Boyden chamber. Serum-free medium containing sSEMA4D and VEGF separately or in combination was placed in the bottom layer as a chemotactic. A solution of 0.1% BSA and 10% FBS was used as a control. HUVECs transfected with shRNA were placed in the upper layer. The control group was used to determine changes in HUVEC migration ability in each group.

### Vasculogenic mimicry analysis

Vasculogenic tube formation was tested using a commercial Matrigel assay kit (BD Biosciences). We coated 24-well culture plates with Matrigel matrix (0.1 ml/well; BD Biosciences) and allowed it to solidify at 37 °C for 30 min. HUVECs and A2780 cells were added to the plates 24 h after transfection. The cells were dissociated into single cells and resuspended at 6 × 10^4^ cells/ml in endothelial basal medium containing 2% fetal calf serum. The cells in each group were then plated at 0.5 ml/well onto the Matrigel surface and incubated at 37 °C. Tube networks were quantified as the total number of pixels (×200) in thresholded images using Image Pro Plus 6.0 software. A total of 3 wells were randomly selected.

### Quantitative RT-PCR

Total RNA was reverse transcribed into cDNA using a reverse transcription kit (Bao Biological Engineering (Dalian) Co., Ltd.) and quantitative real-time PCR was performed on a Bio-Rad CFX96 instrument. The following solutions were successively added into the PCR mixture (20 μl total): 1 μl of cDNA, 10 μl of QIAGEN SYBR Green, 5 μl of RNase-free H_2_O, and 2 μl of upstream and downstream primers. The PCR conditions and procedures were: pre-denaturation at 95 °C for 5 min, 40 cycles of denaturation at 95 °C for 10 s and annealing at 60 °C for 30 s. β-actin levels were used to normalize expression.

### Western blot

Cells were lysed in lysis buffer using the RIPA method. The lysate was subject to SDS-PAGE, electrically transferred onto nitrocellulose membrane and incubated at 4 °C overnight in primary antibody. Membranes were washed with TBST and incubated in secondary antibody for 1 h. The membrane was then washed with TBST and exposed.

### Immunohistochemistry

Human EOC tissues and adjacent noncancerous tissues were collected from the Tianjin Medical University Cancer Institute and Hospital. Informed consent was obtained from each patient. The Ethics Committee of Tianjin Medical University granted ethical approval. Immunohistochemistry was performed according to previously described methods. The sections were pre-treated using microwave irradiation, blocked, and incubated with polyclonal antibodies. The staining intensity was then assessed.

### Data analysis

Statistical analysis was done using SPSS 18.0 statistical software. Data were represented as means and standard deviation. *p* < 0.05 was considered statistically significant.

All experiments were performed in accordance with relevant guidelines and regulations.

## Results and discussion

### Positive correlation between VEGF and SEMA4D expression

**Fig. 1:**
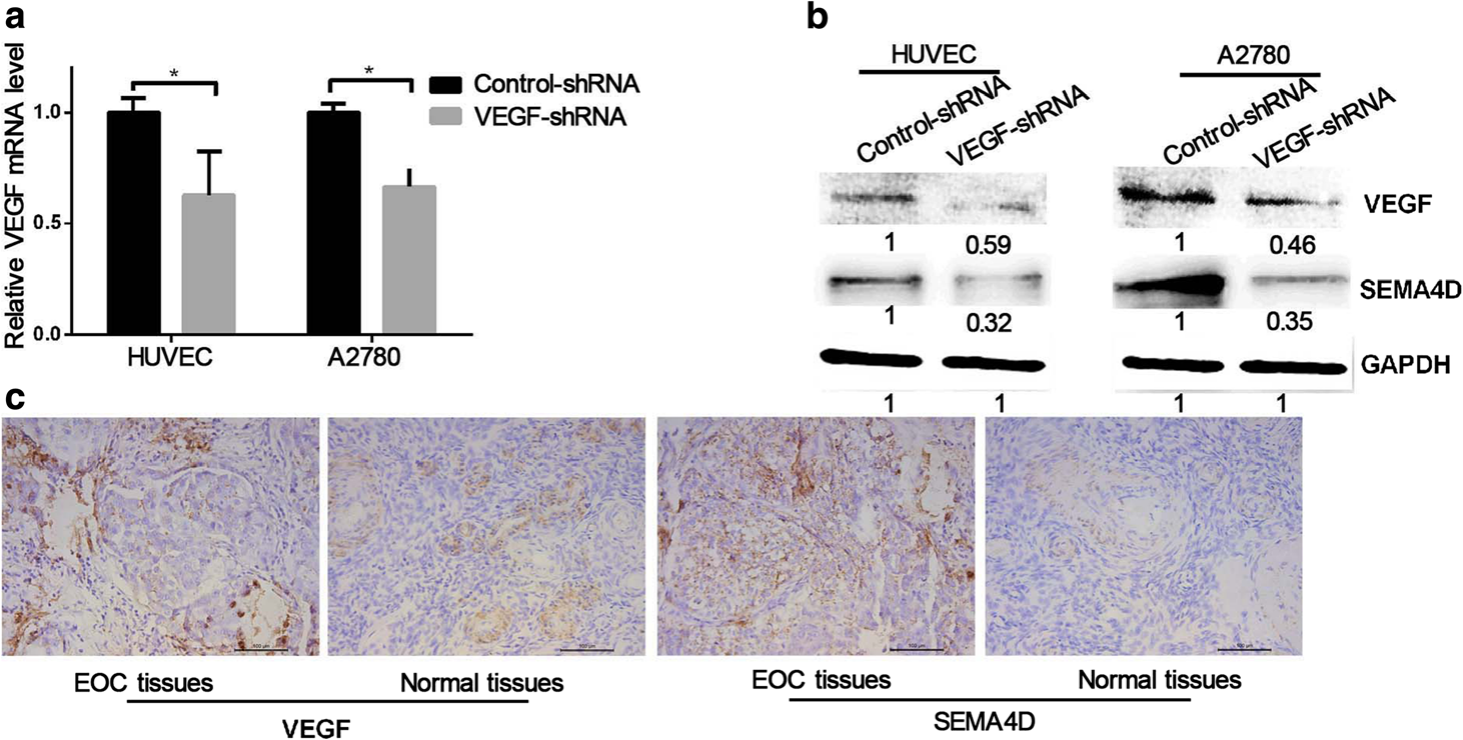
VEGF and SEMA4D expression in EOC cells and tissues. **a** The expression of VEGF in stably transfected cells. β-actin levels were used to normalize expression. **b** The expression of VEGF and SEMA4D in cells transfected with shRNA. **c** The expression of VEGF in EOC tissues and normal tissues

To explore the change in SEMA4D expression when VEGF expression is inhibited in ovarian cancer cells, we constructed a VEGF-knockdown plasmid and screened the stably transfected cells. Quantitative RT-PCR and western blot were used to verify the effectiveness of transfection and stability of the cells (Fig. 1a). Figure 1b shows that the expressions of VEGF and SEMA4D were significantly reduced in these cells compared with the expressions in A2780 cells or HUVECs (by approximately 50% and 40%, respectively).

To explore the relationship between VEGF and SEMA4D in A2780 cells and HUVECs, 124 samples of epithelial ovarian cancer tissues and 20 samples of normal ovarian tissues were subjected to immunohistochemical examination. As shown in Fig. 1c and Table 1, the expression levels of SEMA4D and VEGF in epithelial ovarian cancer tissues were significantly higher than those in normal ovarian tissue. We also applied correlation analysis to the expressions of SEMA4D and VEGF in epithelial ovarian cancer tissues. The results revealed a positive correlation between VEGF and SEMA4D (Table 2).

**Table 1:** VEGF and SEMA4D expression levels in ovarian cancers and normal ovarian tissues

Group	Cases *N*	SEMA4D positive expression *n* (%)	p	VEGF positive expression *n* (%)	p
EOC tissues	124	76 (61.3)	< 0.001	52 (41.9)	< 0.001
Normal ovary	40	4 (10)		4 (10)	

*EOC* Epithelial ovarian cancer

**Table 2:** Relationship between VEGF and SEMA4D expression in EOC

		Cases (*N*)	VEGF expression *n* (%)		ρ	p
			Negative	Positive	0.263	0.002
SEMA4D expression *n* (%)	Negative	48	36 (75.0)	12 (25.0)	0.412	0.000
	Positive	76	36 (47.4)	40 (52.6)		

*EOC* Epithelial ovarian cancer

### Vasculogenic mimicry promoted by the synergistic action of SEMA4D and VEGF

**Fig. 2:**
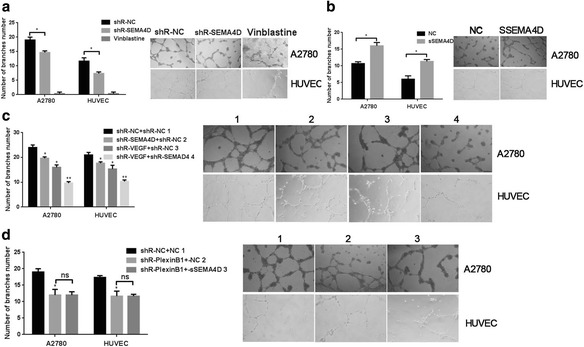
Vasculogenic mimicry (VM) promoted by the synergistic action of SEMA4D and VEGF. **a** VM in SiR-SEMA4D stably transfected cells. **b** VM in the cells with SEMA4Ds. **c** VM in cells co-transfected with SEMA4D and VEGF. **d** The VM formation of A2780 and HUVEC cells when added sSEMA4D and/or shR- PlexinB1. A total of 3 wells were randomly selected

To study the role of SEMA4D in vasculogenic mimicry (VM), cells stably transfected with siR-SEMA4D were constructed and subjected to VM analysis. Figure 2a shows that vessel formation decreased by approximately 40% or 60% when SEMA4D was knocked down in A2780 cells or HUVECs, respectively. We prepared sSEMA4D significantly promoted VM of HUVECs (Fig. 2b).

When HUVECs and A2780 cells were co-transfected with vectors to overexpress SEMA4D and VEGF, this largely suppressed vessel formation (Fig. 2c). When sSEMA4D and VEGF were added to cells stably transfected with We also constructed VEGFR2-shRNA and plexin-B1-shRNA, no suppression recovery was seen in VM (Fig. 2d). These results indicate that SEMA4D and VEGF synergistically promote angiogenesis in HUVEC cells and in cells of the epithelial ovarian cancer line A2780.

### SEMA4D and VEGF synergistically promote migration

**Fig. 3:**
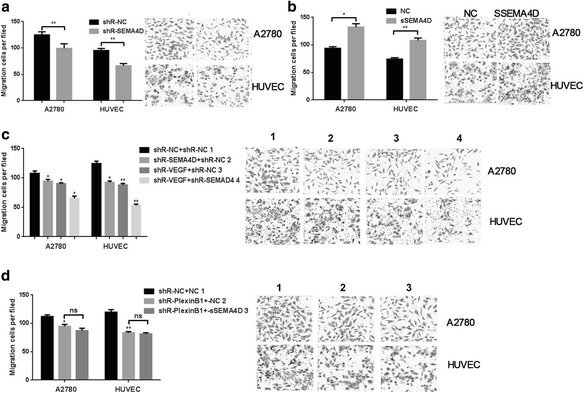
SEMA4D and VEGF synergistically promote migration. **a** Representation of migration in cells stably transfected with siR-SEMA4D. **b** Representation of migration in cells with the addition of SEMA4Ds. **c** Representation of migration in cells co-transfected with SEMA4D and VEGF. **d** The migration of A2780 and HUVEC cells when added sSEMA4D and/or shR- PlexinB1. A total of 3 wells were randomly selected

We further explored the effects of SEMA4D and VEGF on migration, using the transwell migration assay to assess migration ability. As shown in Fig. 3a, migration decreased by approximately 30% or 45% when SEMA4D was knocked down in A2780 cells or HUVECs, respectively.

SSEMA4D were found to significantly promote migration of HUVECs (Fig. 3b). In addition, co-transfection of SEMA4D and VEGF into HUVECs and A2780 cells largely suppressed migration (Fig. 3c). Subsequently, we added sSEMA4D and VEGF to cells stably transfected with VEGFR2-shRNA and plexin-B1-shRNA but found this did not rescue the suppression of VEGFR and plexin-B1 on migration (Fig. 3d). These results indicate that SEMA4D and VEGF synergistically promote the migration ability of epithelial ovarian cancer cells.

**Fig. 4:**
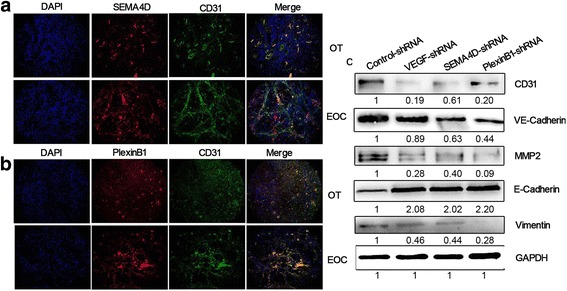
The expression and location of SEMA4D in clinical specimens showing the context of angiogenesis. **a** Immunofluorescence staining showing the expression of SEMA4D and CD31 in EOC and OT (ovarian tissues). **b** Immunofluorescence staining showing the expression of SEMA4D and plexin-B1 in EOC and OT. **c** Western blot revealing the expression of protein in cells transfected with shRNA

To confirm the role of SEMA4D in angiogenesis, we applied immunofluorescence staining to examine the location of SEMA4D in ovarian cancer tissues and normal tissues. We also examined the endothelial cell marker CD31, which targets vascular endothelial cells. Immunofluorescence staining showed that the vascular density in ovarian cancer tissues increased significantly, and that the expression of SEMA4D considerably increased in the endothelial cells in blood vessels. SEMA4D was found to co-localizes with CD31 (Fig. 4a). We also detected the expression and location of plexin-B1 in clinical ovarian cancer tissues and normal tissues. The results indicate that plexin-B1 localizes in the area of endothelial cells (Fig. 4b). SEMA4D and plexin-B1 expression levels were considerably higher in ovarian cancer tissues than in normal tissues.

### The molecular mechanism of VEGF–SEMA4D promotion in angiogenesis

We examined the expressions of the markers of angiogenesis. When VEGF or SEMA4D were inhibited, CD31 expression reduced by 61.1% and 52.8%, respectively. We also found that CD31 expression was inhibited significantly when plexin-B1 expression was inhibited (Fig. 4c). When VEGF, SEMA4D and plexin-B1 were knocked down, the expressions of MMP2 and VE-cadherin, which are involved in the initiation of angiogenesis, were reduced. Finally, when VEGF, SEMA4D and plexin-B1 were knocked down, the expression of E-cadherin reduced, while that of vimentin increased (Fig. 4c). These results indicate that VEGF, SEMA4D and plexin-B1 could inhibit EMT, which could also explain their inhibition of migration.

### Relationship of VEGF–SEMA4D to malignancy and prognosis

**Fig. 5:**
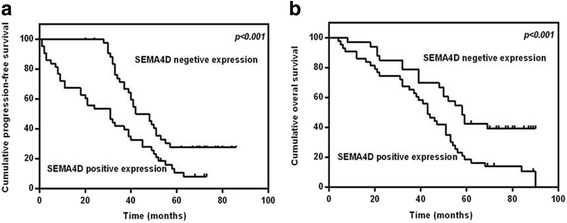
The overall survival and progression-free survival of epithelial ovarian cancer patients with positive and negative expression of SEMA4D. A –Univariate analysis was used to analyse the progression-free survival of EOC patients with positive or negative expression of SEMA4D. B –The overall survival survival of EOC patients with positive or negative expression of SEMA4D analysed by univariate analysis

We further analyzed the correlation between SEMA4D–VEGF and the malignancy of clinical samples. We used the chi-square test to demonstrate that SEMA4D and VEGF expression levels increase in epithelial ovarian carcinomas with a higher degree of malignancy or clinical aggressiveness (Table 3). Univariate analysis indicated that the overall survival and progression-free survival of epithelial ovarian cancer patients with positive expression of SEMA4D were lower than those for epithelial ovarian cancer patients with negative SEMA4D expression (Table 4 and Fig. 5). A multivariate proportional hazards model indicated that SEMA4D is an independent prognostic factor based on the analysis of epithelial ovarian cancer patient prognosis.

**Table 3:** Correlation between VEGF and SEMA4D expression and clinicopathologic characteristics of EOC patients

Variable	Cases (*N*)	SEMA4D positive expression		VEGF positive expression	
		*n* (%)	p	*n* (%)	p
Age			0.078		0.431
≤ 50 years	60	32 (53.3)		23 (38.3)	
> 50 years	64	44 (68.8)		29 (45.3)	
Menopausal status			0.223		0.913
Yes	78	51 (65.4)		33 (42.3)	
No	46	25 (54.3)		19 (41.3)	
Pathologic type			0.709		0.581
Serous carcinoma	80	50 (62.5)		35 (43.8)	
Mucous and others	44	26 (59.1)		17 (38.6)	
Histologic grade			0.000		0.039
G_1_-_2_	49	20 (40.8)		15 (30.6)	
G_3_ or undifferentiated	75	56 (74.7)		37 (49.3)	
FIGO Stage			0.016		0.000
I–II	53	26 (49.1)		8 (15.1)	
III–IV	71	50 (70.4)		44 (62.0)	
LN metastasis			0.017		0.062
No	74	39 (52.7)		26 (35.1)	
Yes	50	37 (74.0)		26 (52.0)	
Residual disease			0.004		0.304
< 1 cm	94	51 (54.3)		37 (39.4)	
≥ 1 cm	30	25 (83.3)		15 (50.0)	
Patients’ response to chemotherapy			0.349		0.010
CR	87	51 (58.6)		30 (34.5)	
PR, SD and PD	37	25 (67.6)		22 (59.5)	
Tumor sensitivity to chemotherapy			0.315		0.006
Platinum sensitive	92	54 (58.7)		32 (34.8)	
Platinum resistant and refractory	32	22 (68.8)		20 (62.5)

**Table 4:** Univariate analysis of OS and PFS in EOC patients

Variable	Cases (*N*)	Media of OS	*y* ^2^	p	Media of PFS	*y* ^2^	p
Age			3.212	0.073		1.830	0.176
≤ 50 years	60	54			41		
> 50 years	64	43			34		
Menopausal status			0.076	0.782		0.069	0.793
Yes	78	48					
No	46	51					
Pathologic type			0.354	0.552		0.002	0.967
Serous carcinoma	80	43			41		
Mucous and others	44	53			36		
Histologic grade			26.047	0.000		5.636	0.018
G_1–2_	49	85			48		
G_3_ or Undifferentiated	75	43			33		
FIGO Stage			32.221	0.000		11.370	0.001
I–II	53	68			49		
III–IV	71	39			33		
LN metastasis			17.484	0.000		12.361	0.000
No	74	56			48		
Yes	50	35			33		
Residual disease			24.872	0.000		5.824	0.016
< 1 cm	94	54			41		
≥ 1 cm	30	35			33		
Patients’ response to chemotherapy			16.060	0.000		5.393	0.020
CR	87	53			40		
PR, SD and PD	37	32			34		
Tumor sensitivity to chemotherapy			10.502	0.001		13.085	0.000
Platinum sensitive	92	53			45		
Platinum resistant and refractory	32	22			10		
VEGF			29.685	0.000		1.945	0.163
Negative	72	58			41		
Positive	52	32			34		
SEMA4D			34.933	0.000		16.541	0.000
Negative	48	NR			49		
Positive	76	41			31		

*FIGO* International Federation of Gynecology and Obstetrics, *NR* not reached, *CR* complete response, *PR* partial response, *SD* stable disease, *PD* progressive disease

VEGF is an important molecule in ovarian cancer and its expression is negatively correlated with prognosis. Orre et al. found that the vascular density in ovarian malignant tumors is significantly higher than that in borderline and benign tumors. In ovarian clear cell carcinoma, the expression of VEGF is significantly correlated with vascular density [21]. Through immunohistochemical analysis of 110 cases of epithelial ovarian cancer, Yamamo et al. found that the VEGF-positive rate in poorly differentiated ovarian cancer specimens was significantly higher than that of well-differentiated ovarian cancer and benign ovarian tumors. ELISA assessment revealed that VEGF levels were significantly higher in the serum of ovarian cancer patients with malignant tumors than in those with borderline and benign tumors. After tumor resection, VEGF levels decreased significantly, whereas tumor recurrence increased again, suggesting that VEGF levels in the blood could be used as prognostic indicators of ovarian cancer [22].

Here, we found that knockdown of VEGF expression led to the inhibition of VM and migration. We also used VEGF to detect the formation of VM and migration in ovarian cancer cells and HUVECs.

Despite the importance of VEGF inhibition in clinical trials, the outcome was not perfect. For example, GOG218 [12] and ICON7 [13] phase III clinical trials showed that chemotherapy plus bevacizumab gave no significant improvement in overall survival for ovarian cancer patients after first-line treatment despite a slight reduction in progression-free survival. Thus, some pro-angiogenic activity may be increased during anti-VEGF therapy. In this study, we found that SEMA4D may participate in the failure of anti-VEGF therapy.

SEMA4D, a member of the SEMA family within the semaphorin superfamily, plays an important role in the nervous system, the immune system, angiogenesis, and tumor invasion and metastasis. We found that SEMA4D is highly expressed in colon, breast and lung cancer tissues and plays an important role in the development of squamous cell carcinoma of the head and neck and the induction of angiogenesis [23]. In the tumor microenvironment, SEMA4D expression plays a critical role in tumor angiogenesis and vascular maturation and increases the tumorigenicity of tumor cells. In cells not expressing SEMA4D, tumorigenicity and tumor metastasis are seriously weakened [24].

We confirmed that SEMA4D promotes the angiogenesis of ovarian cancer cells. SEMA4D is also known to bind to its receptor plexin-B1 to induce and promote tumor angiogenesis via the Met signaling pathway [21]. Here, we found that SEMA4D promotes tumor VM and migration, partly explaining the promotional effects of SEMA4D on angiogenesis. Moreover, we found that the addition of sSEMA4D to the medium of ovarian cancer cells and HUVECs promotes the formation of VM and migration. This ability was largely inhibited when the receptors of VEGF and SEMA4D were knocked down. Furthermore, we found that VEGF and SEMA4D were significantly correlated with the malignant degree of ovarian cancer through data analysis of clinical specimens. SEMA4D can be used as an independent prognostic factor.

A variety of genes and bio-processes are involved in angiogenesis, including CD31, VE-cadherin, MMP2 and EMT. CD31 is a bio-marker for endothelial cells. VE-cadherin is a transmembrane cadherin protein located in the endothelial cell adherence region. It can maintain the vascular permeability and stability of the intravascular environment, which is a starting element in angiogenesis. Hendrix et al. [23] found that VE-cadherin promotes the formation of VM in highly invasive melanoma cells.

The MMP family of proteins plays an important role in remodeling the pericellular environment and degrading the extracellular matrix (ECM) [25]. Cell behaviors such as cell proliferation, adhesion, migration, invasion, apoptosis and host defense are all related to the MMPs [22]. MMP2 is necessary for classical angiogenesis [24]. The EMT process could mimic the onset of angiogenesis, which is an important molecular mechanism.

Here, we found that knockdown of VEGF, SEMA4D and plexin-B1 could suppress the expression of CD31, VE-cadherin and MMP2, and the process of EMT which indicated that VEGF, SEMA4D and plexin-B1 could promote the onset of angiogenesis.

Our research aims to define the role of SEMA4D in anti-VEGF failure. We have shown that SEMA4D coordinates with VEGF during angiogenesis via plexin-B1 in EOC. This finding is significant for improving our understanding of chemotherapy resistance and the prognosis for patients with advanced epithelial ovarian cancer, for whom traditional means of treatment have proven ineffective.

## Conclusions

VEGF and SEMA4D have synergistic effects on the promotion of angiogenesis in epithelial ovarian cancer. This provides a reference for therapy that should be investigated further.

## Abbreviations

BSA: Bovine serum albumin
CD31: Platelet endothelial cell adhesion molecule-1
EOC: Epithelial ovarian cancer
FBS: Fetal bovine serum
HUVEC: human umbilical vein endothelial cells
MMP2: Matrix metalloprotein 2
MT1-MMP: Matrix metalloprotein 14
SEMA4D: Semaphorin 4D
VE-cadherin: Vascular endothelial cadherin
VEGF: Vascular endothelial growth factor
VM: Vasculogenic mimicry
